# No- or Low-Content Copper Compounds for Controlling *Venturia oleaginea*, the Causal Agent of Olive Leaf Spot Disease

**DOI:** 10.3390/plants13050600

**Published:** 2024-02-22

**Authors:** Leen Almadi, Samer Jarrar, Layth Sbaihat, Tahreer Issa, Michele Tucci, Chiaraluce Moretti, Roberto Buonaurio, Franco Famiani

**Affiliations:** 1 Department of Agricultural, Food and Environmental Sciences, University of Perugia, Via Borgo XX Giugno 74, 06121 Perugia, Italy; leenalmadi1993@gmail.com (L.A.); chiaraluce.moretti@unipg.it (C.M.); roberto.buonaurio@unipg.it (R.B.); 2 Faculty of Agriculture and Natural Resources, Nablus University for Vocational and Technical Education (NU-VTE), Nablus P400, Palestine; 3 Department of Biology and Biotechnology, Faculty of Science, The Arab American University (AAUP), Jenin P.O. Box 240, Palestine; layth.sbaihat@aaup.edu; 4 Independent Researcher, Palestine; eng.tahreer.to@gmail.com; 5 CIHEAM-Bari—Centre International de Hautes Etudes Agronomiques Méditerranéennes, 70010 Bari, Italy; tucci@iamb.it

**Keywords:** azoxystrobin, copper fungicides, difenoconazole, *Olea europaea* L., olive leaf spot, Palestine, peacock eye disease

## Abstract

The efficacy of using a synthetic (azoxystrobin + difenoconazole), copper-based (copper oxychloride) and low-content copper compound (copper complexed with gluconate and lignosulphonate) fungicides for controlling *Venturia oleaginea*, the causal agent of olive spot disease, was evaluated in an olive (cv. Nabali) orchard located in the Kafr Qud area (Palestine) in 2017–2018. Treatments were applied at three different times (February, April, and August). In January 2017, at the beginning of the experiment, about 90% of the leaves grown in 2016 were infected. Defoliation was determined by counting the leaves on the labeled branches initially and then periodically. It increased gradually in both the control and treated trees, but those treated with azoxystrobin + difenoconazole or with copper complexed with gluconate and lignosulphonate showed a slower defoliation rate. During 2017, new shoots grew and new leaves developed. All treatments reduced the drop of new leaves with respect to the control, with positive effects on the reproductive activity (inflorescence growth and yield). Overall, all treatments significantly reduced the disease, thus indicating the possibility of greatly reducing infections if treatments are regularly applied each year, also with traditional (copper-based) fungicides. Due to their capability of penetrating inside the vegetative tissue, azoxystrobin + difenoconazole or copper complexed with gluconate and lignosulphonate reduced/slowed down the drop of infected leaves. The use of these fungicides is therefore particularly recommended when olive leaf spot disease is severe. The use of low-content copper compounds allows the amount of metallic copper used for the treatments against *V. oleaginea* to be greatly reduced.

## 1. Introduction

Olive leaf spot (OLS) caused by *Venturia oleaginea* (Castagne) Rossman & Crous (the anamorph: *Spilocaea oleaginea*) is a dangerous olive disease widespread in the Mediterranean regions where it can cause severe yield losses [1,2,3,4]. It is characterized by premature defoliation, twig dieback, and, when infections are very severe, reduced flowering and fruit set [5,6,7,8]. The pathogen can also attack the fruit, causing delayed ripening, decreased oil content and unacceptable blemishes on table olives [5,9]. There is scarce information on the effects of the disease on yield, and more knowledge on this aspect would be very useful. OLS disease is one of the main concerns in olive cultivation in all olive-growing areas [4,8,10,11], and a very different susceptibility to the disease has been observed among olive varieties [12,13,14]. Fungal sporulation and conidia germination and infections are favored by wet weather conditions, which are prevalent in some areas or that derive from rainy seasons, and/or dense vegetation causing an increase in relative humidity inside the canopy due to short distances between trees and/or incorrect pruning, especially in non-aerated areas [11,15,16,17,18].

The heavy damage that OLS disease can cause, especially in susceptible cultivars and wet environments, has been a great stimulus for carrying out studies on treatments for its control. Information on the negative effects of OLS disease on yield is very limited. However, reductions of 20–30% have been estimated [8,15], whereas no information is available on the possible effects of the disease on oil quality. The application of copper-based fungicides is currently the main method for controlling OLS disease in all olive-growing countries.

Several studies have been carried out to evaluate the efficacy of copper compounds and other fungicides, such as dodine [19,20,21,22,23], systemic triazoles: hexaconazole and tetraconazole [23,24], azoxystrobin (systemic fungicides of the strobilurin family) + difenoconazole (another systemic triazole fungicide) [25], and trifloxystrobin (strobilurin family) + tebunconazole (triazole family) [26]. Studies have also included the use of natural compounds and substances able to induce systemic acquired resistance [22,27]. However, there have been no conclusive results because the efficacy of the different compounds/fungicides depends on factors, such as the application time, the degree of leaf infection at the time of treatment and the interaction with the environmental conditions.

In the last few years, increasing concern on the use of copper-based fungicides has arisen because copper is a heavy metal that can accumulate in the soil, creating environmental problems. Consequently, the European Union has limited the use of metallic copper to 28 kg/ha of over a period of 7 years (on average 4 kg/ha/year), and further limitations are likely in the near future. This has pushed researchers to find fungicides with a low copper content or fungicides based on other active ingredients in order to reduce/eliminate copper for controlling OLS disease. Dodine is among the no-copper-containing fungicides, but its use is often restricted. Indeed, in European countries, such as Italy, protocols for integrated pest management specify that only one dodine treatment/year is allowed. Therefore, it is interesting to look for other compounds that can eliminate the use of copper or greatly reduce it. In this regard, azoxystrobin + difenoconazole and fungicides with low copper content seem promising, but results on their use are not conclusive [25,28]. Therefore, further studies are necessary to obtain a better understanding about the efficacy and the best conditions for the use of synthetic or low-copper-containing fungicides that have been proposed for OLS disease control.

The aim of the present study was to evaluate the use of different fungicides, both synthetic (i.e., azoxystrobin + difenoconazole) and copper-based (with different amounts of copper), for controlling OLS disease in olive. The experiment was carried out in Palestine, in an area very favorable to the disease (Jenin region), in an olive orchard of the cultivar Nabali, which is very susceptible to *V. oleaginea*.

## 2. Results

### 2.1. Climatic Data

**Figure 1:**
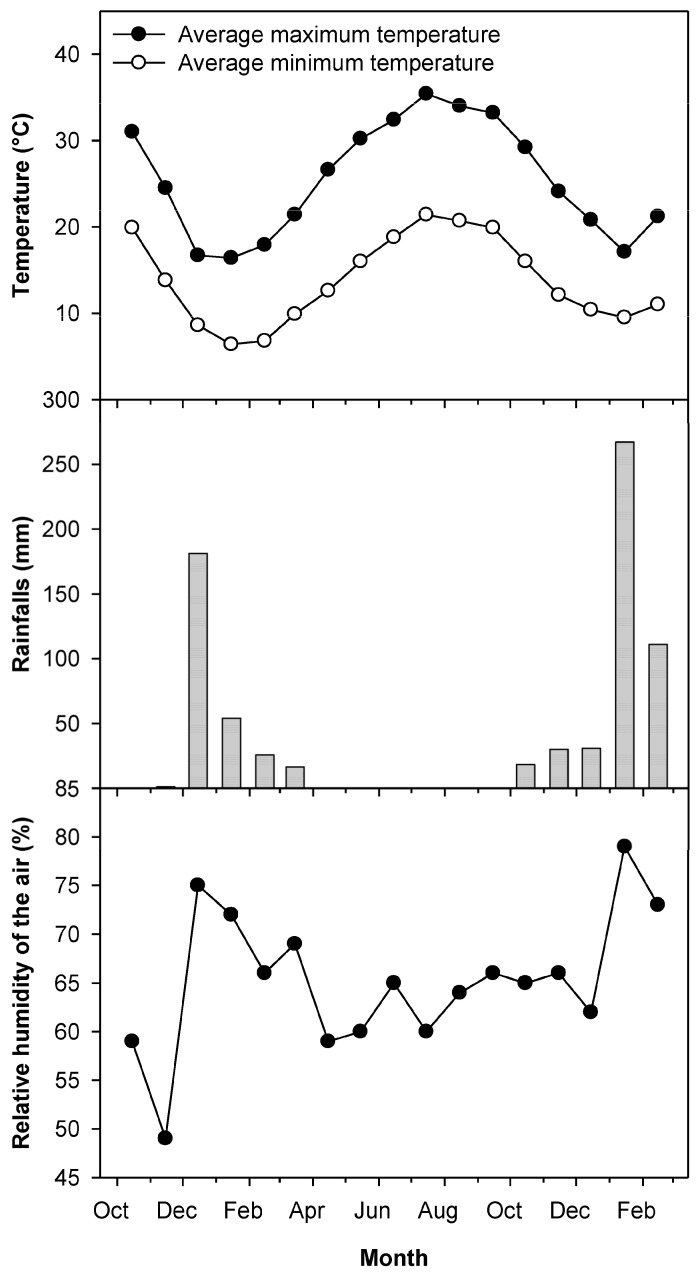
Climatic data recorded from October 2016 to February 2018 from the Jenin weather station located near the Kafr Qud farm: average monthly maximum and minimum temperatures and relative humidity of the air and monthly cumulated rainfalls.

Maximum and minimum temperatures showed the lowest and highest values in January and August 2017, respectively (Figure 1). Rainfalls were concentrated in the periods December–March 2016–2017 and October–February 2017–2018 (Figure 1). The relative humidity (RH) was high in the periods December–March 2016–2017 and January–February 2018 and showed values around 60–65% in the other months of 2017 (Figure 1).

### 2.2. Effects of OLS Disease on Leaves Grown in 2016 and Still Present at the Beginning of 2017

In order to verify the efficacy of no- or low-content copper compounds in controlling *V. oleaginea*, a common copper-based fungicide (copper oxychloride), in comparison with a systemic product based on azoxystrobin + difenoconazole, and a particular formulation of a low-content copper compound complexed with gluconate and lignosulphonate were used on cv. Nabali olive trees. The experiment started in January 2017, when the leaves grown in 2016 were present. The symptomatic leaves were 60–70% of the total (Table 1), while the asymptomatic but infected ones were 24–31% of the total. This means that about 90% of the leaves grown in 2016 were infected (Table 1).

**Figure 2:**
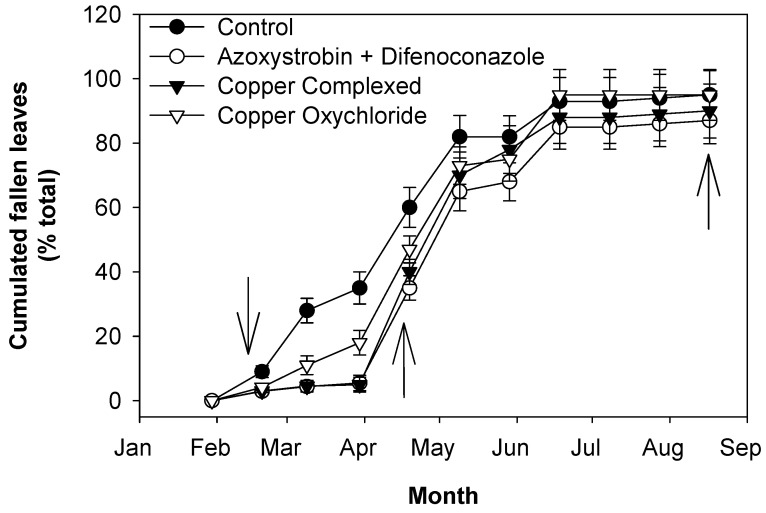
Effects of fungicide treatments on the control of peacock eye: defoliation caused by the disease on leaves grown in 2016 and still present in 2017. Arrows indicate treatments with fungicides. Bars represent the standard error.

Since infected leaves fall, defoliation was evaluated by counting the remaining leaves on the tree with respect to those which were present at the beginning of the experiment. The application of treatments determined changes in the drop patterns of leaves. Defoliation, which was determined by counting the leaves on the labeled branches at the beginning of the experiment and then periodically (about every 20 days), increased gradually in both control and treated trees. Those treated with azoxystrobin + difenoconazole or with copper complexed with gluconate and lignosulphonate showed less defoliation than the control. Plants treated with copper oxychloride, especially between the first treatment in mid-February and late March (Figure 2), showed lower protection than those treated with azoxystrobin + difenoconazole or with copper complexed with gluconate and lignosulphonate, and then the differences decreased.

### 2.3. Effects of OLS Disease on Leaves Grown in 2017

**Figure 3:**
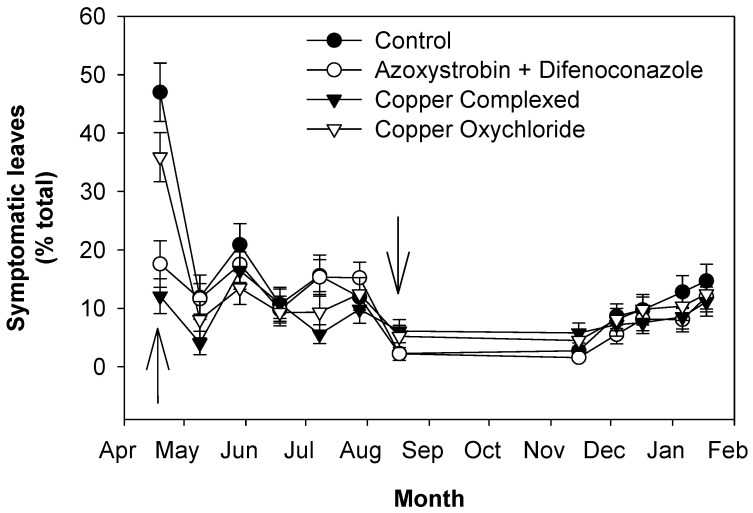
Effects of fungicide treatments on the control of peacock eye: evolution of new symptomatic leaves (grown in 2017). Arrows indicate treatments with fungicides. Bars represent the standard error.

**Figure 4:**
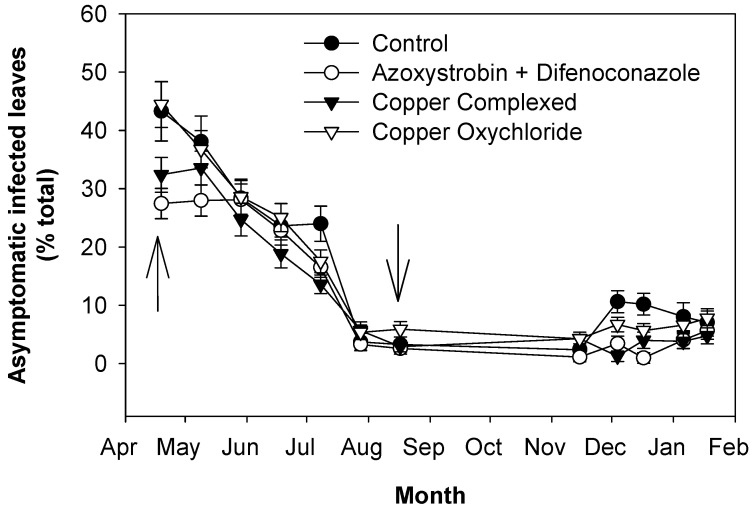
Effects of fungicide treatments on the control of peacock eye: evolution of asymptomatic infected leaves (grown in 2017). Arrows indicate treatments with fungicides. Bars represent the standard error.

In order to monitor the level of infection in the new leaves, new grown shoots and leaves developed up to July 2017 were examined. The first observations on new leaves were performed in mid-April at the time of applying the second treatment. In the few leaves present at that time, symptomatic and asymptomatic infected leaves were detected, especially in the control trees and trees treated with copper oxychloride (Figure 3 and Figure 4). Then, shoots continued to grow, and symptomatic and asymptomatic infected leaves decreased in all the treatments. Both symptomatic and asymptomatic infected leaves reached the lowest values in August and remained at these low levels up to November, when, in all cases, there was an increase, with the control trees showing the highest values.

**Figure 5:**
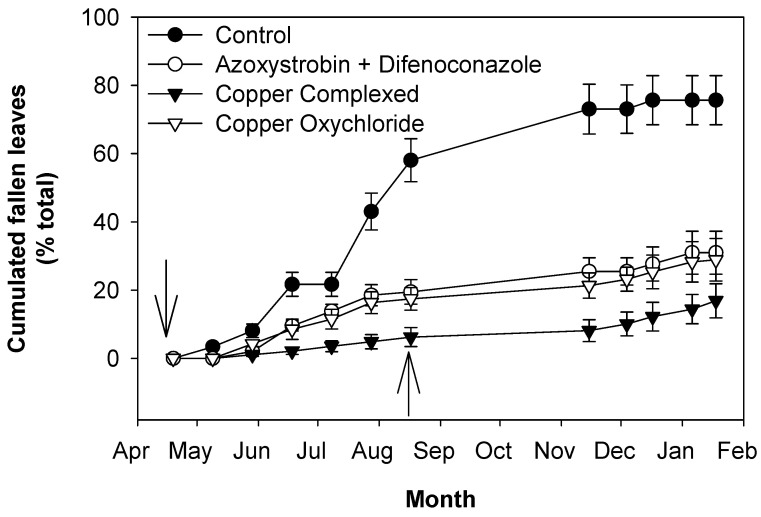
Effects of fungicide treatments on the control of peacock eye: defoliation caused by the disease on leaves grown in 2017. Arrows indicate treatments with fungicides. Bars represent the standard error.

The control trees showed an earlier and intense drop of leaves, reaching about 20% in June–July, 60% in August, 70% in November and 75% in December–January (Figure 5, Table 2). The treated trees showed a much slower increase in defoliation (Figure 5; Table 2). All the treated trees showed an increase in defoliation up to August; then, no significant changes were observed up to November at which time there was an increase up to the end of the experiment (January 2018). The treatments determined different degrees of defoliation: trees treated with copper complexed with gluconate and lignosulphonate reached values around 5% in August and remained lower than 10% up to November, reaching values around 17% in January 2018, while trees treated with copper oxychloride showed values around 17% in August, 21% in November and 31% in January 2018. Azoxystrobin + difenoconazole reached values of 19% in August, 25% in November and 31% in January 2018.

At the end of the experiment, on the treated trees, the non-dropped leaves were 69–83% of the total leaves developed during 2017 and around 26% on the control trees (Table 2).

Table 3 reports the percentages of symptomatic and asymptomatic leaves with respect to those still on the canopy (i.e., excluding the leaves that had dropped), not with respect to the total leaves developed, which are those on the canopy + those that had fallen.

### 2.4. Effects of OLS Disease on Inflorescence Development and Olive Yield

**Figure 6:**
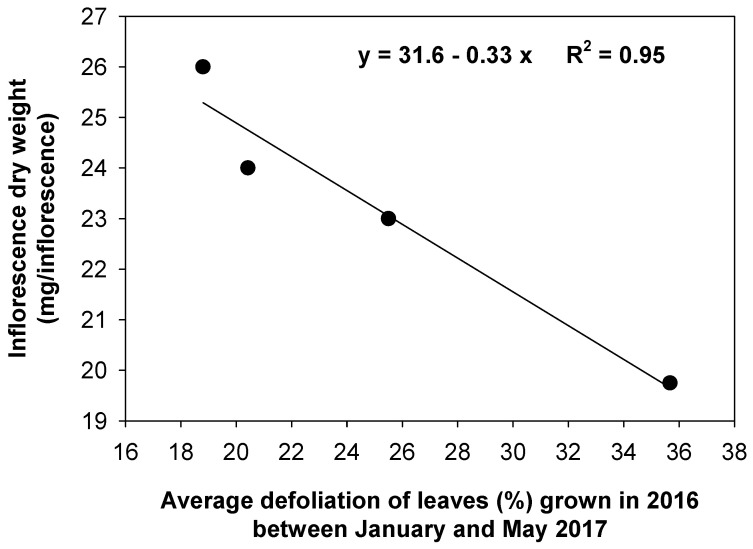
Relationship between the average defoliation of leaves grown in 2016 between January and May 2017 and the inflorescence dry weight (at the white stage of inflorescence development).

**Figure 7:**
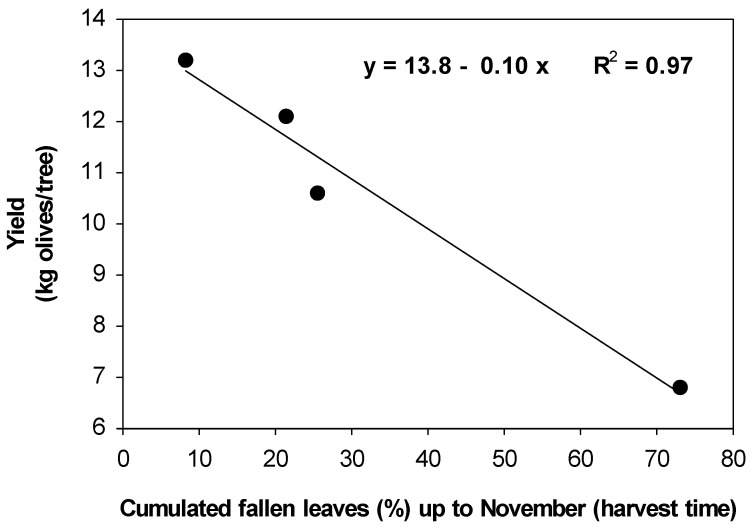
Relationship between the cumulated fallen leaves (grown in 2017) up the November (harvest time) and the yield (olives) of the trees.

Both the dry weight of the inflorescences and olive yield were higher in the treated trees than in the control trees (Table 4). There were significant and negative linear relationships between the average drop between January and May 2017 of leaves grown in 2016 and the weight of inflorescences (Figure 6) and between the cumulated drop, up to November (harvesting time), of leaves grown in 2017 and the yield (Figure 7).

## 3. Discussion

Although the environmental conditions were characterized by low amounts of rainfall and dry conditions for most of the growing season, a high disease incidence (about 90%) was recorded at the beginning of the experiment in January 2017. This is probably due to (i) the high susceptibility of the cultivar Nabali to *V. oleaginea* [8,10,29]; (ii) the favorable climatic conditions for the disease [17,30,31] (i.e., relatively high values of rain and RH) recorded at the end of autumn and beginning of winter in 2016 (see Figure 1); and (iii) the fact that no treatments against OLS disease were applied to the trees in 2016.

In general, fungicide treatments applied in mid-February and mid-April, compared to the control, tended to slow down the drop of leaves grown in 2016, especially those with azoxystrobin + difenoconazole or copper complexed with gluconate and lignosulphonate, which showed the lowest values of defoliation (Figure 2). The high level of defoliation is the result of the high disease incidence at the beginning of the experiment and the fact that infected leaves drop [19,23,32]. The reduction in the defoliation rate in all treated trees may be due, at least in part, to the prevention of/reduction in the attack of the remaining healthy leaves due to the application of the fungicides. Moreover, since azoxystrobin + difenoconazole or copper complexed with gluconate and lignosulphonate are able to penetrate the leaves, they had a curative effect, which was particularly evident in leaves with the mildest infections.

Regarding the new vegetation in 2017, there was a continuous growth of new shoots up to July. The new leaves on the shoots showed a relatively high disease incidence in the first observation in April, especially in the control and copper-treated trees (Figure 3 and Figure 4). These infection patterns (both symptomatic and asymptomatic) can be explained by considering that the first new leaves grew between the first treatment and the second one, and so they were not directly covered by treatments. Afterward, the April treatments were performed, new leaves grew and the climatic conditions were drier and hotter and thus less favorable to the disease [30,31,33]. All these factors determined a decrease in the percentages of symptomatic and asymptomatic infected leaves, especially in the treated trees, up to August. The decrease in these categories of infected leaves was in part also due to the start of their drop in May, which was intense in the control trees, especially in July and August, whereas in the treated trees, it was much less intense, especially in those treated with copper complexed with gluconate and lignosulphonate. Then, defoliation continued to increase, at a much lower rate, up to November in the control trees, whereas in the treated ones, it did not increase significantly. From November on, in all trees, infections and defoliation increased up to the end of the experiment (January 2018). From the infection and defoliation patterns of the control and treated trees, it can be observed that treatments with fungicides to control peacock eye disease on new leaves play a fundamental role. Climatic conditions also contributed but to a much lesser degree. Indeed, periods with higher humidity and temperature suitable to peacock eye (i.e., from November 2017 to January 2018) favored the increase in the disease in both the control and treated trees, but in the latter, the increases were not as great as in the control.

The greater growth of inflorescences in the treated trees may be the result of the lower degree of defoliation at the end of winter to the beginning of spring of leaves grown in 2016 (Table 4, Figure 2), when inflorescences were growing. Likely, the higher presence of leaves promoted a higher production of assimilates with photosynthesis, and this contributed to inflorescence growth.

Likewise, the higher olive yield in the treated trees may be the result of their lower defoliation throughout the 2017 season (Table 4, Figure 5).

The effects on both inflorescence growth and yield caused by defoliation are also greatly supported by the significant linear and negative relationships between the degree of defoliation and inflorescence weight or yield (Figure 7). As the fruit weight was similar for all treatments (Table 4), the higher yield of the treated trees was due to a higher number of fruits that reached ripening. Therefore, it appears that the effects on yield were more mediated by the number of fruits at harvest than by differential fruit growth.

Overall, the results showed significant effects of the treatments in reducing the effects of disease (especially defoliation), particularly in the first part of the season (up to August). Then, the treatments applied in August blocked the infections in the treated trees up to the end of the vegetative season (November). This was important because the larger number of leaves on the treated trees enabled better growth of inflorescences and higher yields due to a higher number of fruits reaching ripening.

The fungicides based on azoxystrobin + difenoconazole or copper complexed with gluconate and lignosulphonate slowed down the drop of infected leaves grown in 2016 and reduced the first attacks in leaves grown in 2017. This is probably due to the fact that they are able to penetrate the leaves, enabling more efficient control of the disease and longer persistence because they are not washed away by rain. Overall, in the leaves grown in 2017, the traditional copper fungicide (copper oxychloride) gave good control of the disease.

It is very important to note that the control trees, which were not treated, at the beginning of the successive year (2018), had considerably fewer leaves than the treated ones, and the percentage of healthy leaves with respect to the total was around 14%, a percentage similar to that (10%) at the beginning of 2017. In the treated trees, the number of leaves still on the canopy was much higher, with the percentage ranging from 69% to 83% (Table 3 and Figure 5).

The effectiveness of traditional copper-based fungicides has been observed in several previous studies [10,11,19,20,23] and was confirmed here. This contributes to explaining why, to date, the application of copper-based fungicides is still by far the main method to control *V. oleaginea* in all olive-growing countries.

The results of the use of azoxystrobin + difenoconazole are in line with those obtained by Zuffa and Ricci [25]. In a previous study, the use of difenoconazole alone did not produce remarkable effects [20], indicating that the combination with azoxystrobin makes the mix more effective.

Both azoxystrobin + difenoconazole and copper complexed with gluconate and lignosulphonate indicate that copper can be significantly reduced. This is a very important aspect considering that the use of copper, which is a heavy metal, must be reduced to comply with European regulations. It is important to note that the use of copper complexed with gluconate and lignosulphonate allows the amount of copper used for each treatment to be reduced five times with respect to the amount with copper oxychloride. Moreover, these compounds are also interesting from a toxicity point of view because, compared to copper oxychloride, the one with a reduced copper content greatly reduces the impact of such a heavy metal on the environment and farmers’ health, while for azoxystrobin + difenoconazole, the label reports risks for the environment and farmers’ health that are substantially similar to those of copper oxychloride.

To develop a good strategy for the control of OLS, the presence of the disease should be monitored by using the NaOH test [11]. The percentage of infected leaves should be less than 20%. This can be used as a threshold because experiments that evaluated artificial defoliation showed that a reduction in leaf surface of this amount can be compensated for by an increase in the photosynthetic activity of the remaining leaves [34]. Therefore, when the percentage of symptomatic + asymptomatic infected leaves goes beyond this value, treatments should be applied to keep the disease under control. From the results of our study, all the fungicides tested could be used for this purpose.

## 4. Materials and Methods

### 4.1. Location and Characteristics of the Experimental Field

The experiment was carried out in 2017–2018 in the northern part of the West Bank (WB) in Palestine, where the climate is characterized by a long, hot, dry summer and a short, cool, rainy winter. The experiment was carried out on a farm located in the Kafr Qud area, in the Jenin district, which is the main Palestinian area for olive production and where OLS disease is present at a high incidence and severity. The olive trees and soil in the olive orchard were uniform. Conventional cultivation techniques are used, and soil management is performed by applying tillage three–four times per year. Fertilization is carried out by using animal manure, and pruning is conducted every two years.

### 4.2. Fungicides and Time of Application

The treatments were carried out using copper oxychloride, a common copper-based fungicide, a systemic fungicidal mixture containing azoxystrobin + difenoconazole and a particular formulation of a low-content copper compound capable of penetrating the plant tissues as copper is complexed with gluconate and lignosulphonate. Non-treated trees were used as the control.

Copper oxychloride fungicide (Antracnol^®^, Bayer CropScience, Leverkusen, Germany), which contains 37% metallic copper, was used at a dosage of 300 g/hL of water.

The fungicide azoxystrobin 200 g/L (20%) + difenoconazole 125 g/L (Amistar Top^®^, Basel, Switzerland) was used at a dosage of 40 mL/hL of water. Copper complexed with gluconate and lignosulphonate (Disper Cu Max, Eden Agro S.L., Alicante, Spain) contains 14% metallic copper and was used at a dosage of 125 g/hL of water.

The treatments were applied on a dry day, and care was given to distribute the fungicides uniformly over the entire canopy of each treated tree. The treatments were applied in three periods: mid-February, mid-April and mid-August.

### 4.3. Experimental Design

The experiments were carried out according to a completely randomized design, using three trees (three replicates) per treatment. Non-treated trees were used as the control. On each tree, 16 branches were selected and labeled: 8 in the upper and 8 in the lower portions of the canopy. At the beginning of the trial (January 2017), leaves grown in 2016 were on the branches, whereas those grown in 2015 had all fallen off. The leaves grown in 2016 were easily recognized due to the presence of buds corresponding to their insertion on the shoot.

### 4.4. Field Observations

The number of leaves grown in 2016 was recorded at the beginning of the trial. The leaves were periodically counted (about every 20 days) to determine the number of those which had fallen (defoliation) during the season. The defoliation was expressed as the percentage of leaves fallen during the season with respect to the total number of leaves counted in January (the beginning of the experiment).

Observations were also carried out on the new leaves developed during the 2017 season. About every 20 days, the number of leaves were counted, and the symptomatic ones were identified (affected by OLS disease and showing classical lesions).

Just before flowering (the white stage of development), 20 inflorescences per tree were collected, and their length was measured. This was conducted at the National Agricultural Research Center (NARC) in the Department of Plant Production and Protection (Qabatiya/Jenin).

In November, during harvest, the fruit weight (samples of 100 drupes per tree) and the yield were recorded.

### 4.5. Laboratory Observations

The level of infection on asymptomatic leaves grown in 2017 was also determined on the same dates as the labeled branches, using leaves (about 100 leaves per tree) collected from parts of the canopy close to the labeled branches. The leaves were immersed in one liter of a 5% NaOH solution warmed to 65–70 °C [11]. After 2.5 min, the leaves were removed from the NaOH solution, and the number of spots that appeared, which indicates lesions caused by the pathogen, was recorded.

### 4.6. Leaves Grown in 2017: Percentages of Defoliation and Infected Leaves

On the leaves grown in 2017, percentages of defoliation, symptomatic and asymptomatic infected leaves and asymptomatic non-infected leaves were recorded with respect to the total number of leaves produced up to the date of each observation in the field. That is, the total number of leaves consisted of those still on the shoots + those fallen off up to each date of observation.

### 4.7. Climatic Data

The climatic data were recorded at the Jenin weather station located near the Kafr Qud farm. The parameters were as follows: temperature (maximum and minimum), relative humidity of the air and rainfall.

### 4.8. Statistical Analysis

Data are presented as means ± standard error and/or were statistically analyzed by ANOVA according to a completely randomized design, and the averages were compared by using the Student–Newman–Keuls test.

## 5. Conclusions

The results confirmed the very high susceptibility of the cultivar Nabali to *V. oleaginiea*.

All the fungicide treatments were able to promote the reproductive activity of the trees, which is inflorescence growth and olive yield.

The significant reduction in infection obtained after one year of fungicide applications is very encouraging because it indicates that treatments can reduce disease damage also in the years in which OLS is highly present on olive leaves (like the present study) and suggests the possibility of greatly reducing the infections if treatments are regularly applied each year, along with traditional (copper-based) fungicides.

The results indicate the importance of monitoring for infections also using the NaOH test to maintain the percentage of infected leaves at values lower than the 20% threshold.

Due to the ability to penetrate the vegetative tissue, azoxystrobin + difenoconazole and copper complexed with gluconate and lignosulphonate can reduce/slow down the drop of infected leaves. Their use is therefore particularly recommended when disease severity is high. These fungicides also enable the amount of metallic copper used for the control of peacock eye in olives to be reduced significantly.

## Figures and Tables

**Table 1:** Symptomatic and asymptomatic infected leaves at the beginning of the experiment (January 2017).

Treatment	Symptomatic Leaves (%Total)	Asymptomatic Infected Leaves (%Total)	Symptomatic + Asymptomatic Infected (%Total)
Control	68.1 a	24.6 a	92.7 a
Azoxystrobin + difenoconazole	66.3 a	25.6 a	91.9 a
Copper oxychloride	58.8 a	29.1 a	87.9 a
Copper complexed with gluconate and lignosulphonate	59.4 a	31.3 a	90.7 a

In each column, means followed by the same letter are not significantly different at *p* ≤ 0.05.

**Table 2:** Effect of treatments on the fate of all leaves developed in the 2017 season determined at the end of the experiment (January 2018).

Treatment	Dropped Leaves (%Total)	Symptomatic Leaves (%Total)	Asymptomatic Infected Leaves (%Total)	Non-Dropped Leaves (%Total)
Control	75.7 a	14.7 a	7.1 a	26.3 c
Azoxystrobin + difenoconazole	31.0 b	12.0 a	5.7 a	69.0 a
Copper oxychloride	28.9 bc	12.5 a	7.8 a	71.1 a
Copper complexed with gluconate and lignosulphonate	16.9 c	11.2 a	4.8 a	83.1 a

In each column, means followed by the same letter are not significantly different at *p* ≤ 0.05.

**Table 3:** Effect of treatments on leaves developed in the 2017 and still present in the canopy at the end of the experiment (January 2018).

Treatment	Symptomatic Leaves (%Total)	Asymptomatic Infected Leaves (%Total)	Symptomatic + Asymptomatic but Infected (%)	Asymptomatic Non-Infected Leaves (%)
Control	55.9 a	30.0 a	85.9 a	14.1 b
Azoxystrobin + difenoconazole	17.4 b	8.3 b	25.7 b	74.3 a
Copper oxychloride	17.6 b	11.0 b	28.6 b	71.4 a
Copper complexed with gluconate and lignosulphonate	13.5 b	13.5 b	27.0 b	73.0 a

In each column, means followed by the same letter are not significantly different at *p* ≤ 0.05.

**Table 4:** Effects of treatments on inflorescence growth, fruit weight and olive yield.

Treatment	Inflorescence Dry Weight (mg/Inflorescence)	Fruit Weight (g/Fruit)	Yield (kg/Tree)
Control	19.8 c	2.40 a	6.8 a
Azoxystrobin + difenoconazole	26.0 a	2.55 a	10.6 b
Copper oxychloride	23.0 b	2.60 a	12.1 b
Copper complexed with gluconate and lignosulphonate	24.0 b	2.55 a	13.2 b

In each column, means followed by the same letter are not significantly different at *p* ≤ 0.05.

## Data Availability

No new data were created or analyzed in this study. Data sharing is not applicable to this article.
